# The Untruthfulness of Patients

**Published:** 1909-04-03

**Authors:** 


### THE GENERAL PRACTITIONER'S COLUMN.

[Contributions to this Column are invited, and if accepted will be paid for.]

### THE UNTRUTHFULNESS OF PATIENTS.

In one of his inimitable sermons, the eloquent Father Fuller is at some pains to discuss the varieties of untruthfulness. The enumeration of these shades and degrees of lying are in a way as amusing and as practically interesting as Touchstone's list of the species of insults in the giving of the lie. There be those that lie because they will not, those that lie because they cannot, and those that lie because they may not speak truly, and with each and all of these varieties the practitioner has made acquaintance to his great distress of mind, and no less great disturbance of temper. We all know of the untruthfulness of patients, and at some period or other we have all of us had cause to regret that (to quote our Elizabethan preacher once more) men hold not dearer the verisimilitude of fact to the despising of its counterfeits and shame.

In hospital days there loomed the black-listed "chronic" whose wearying catalogue of symptoms would have served the average novelist for a copy of the popular brain fever, or the patient who gorges forbidden fruit on visiting day and denies the overwhelming evidence of his own excreta. In private practice the list can be greatly extended, and every practitioner will be able to furnish examples of instances in which the lie direct, the lie prevaricate, or the lie by intention on the part of his patient has made him stumble, and some in which it has even produced the cousin german to disaster. The full significance of the importance of telling the truth, the student only learns to appreciate when he is alone, fighting his first case as a "locum" or assistant. In private practice, rightly or wrongly, one has to depend to a much greater extent than in hospital practice on what the patient says. As students we are told, by men who have for the most part never been in private general practice, that the statements made by a patient should be disregarded until verified by an accurate examination. As solitary workers in our daily round we learn that hospital methods of minutely examining each case are absolutely out of the question. No private practitioner can afford to waste half an hour or more in making blood counts, or as much time in searching for gonococci. Nor is it possible in private practice to carry out in its entirety the strict personal examination such as the patient in a hospital ward is subjected to. The practitioner has to accept what the patient tells him and to guard himself against error by an intuitive perception of perversions and against loss of temper by remembering that many patients love to indulge in morbid exaggeration.

To a large extent patients lie because they cannot speak the truth. The other two classes may be easily disposed of. Those that will not are usually exposed with facility, and the very small section of those that may not present no great difficulty, at least to the general practitioner. But there is a very large class of patients who cannot speak the truth. Their bent is towards exaggeration, the suppression of fact and the suggestion of the fanciful. They are an interesting class, and in a way we should be grateful for their existence, for they present us with a constant reminder of the fact that imagination is not yet extinct in these isles. The hysteric stands at the head of the list, and we have long since condoned his lying, just as we have condoned the kleptomaniac's thieving, and the epileptic's murdering. Second-class untruthfulness, to abide by Fuller's classification, in the majority of cases is not so much an immorality as a lesion. Many of our patients cannot speak the truth; either through lack of courage or through excess of self-love, they must lie, exaggerate, suppress, and dissemble. It is useless to appeal to their better feelings; to tell them that they are placing their health in jeopardy by treating their medical attendant like a man whose eyes have to be dust-dimmed. They are incapable of recognising these facts, and they will continue to stick to their versions till the end of the chapter, even though their untruthfulness may be made "transparent to the world and trebly exposed." With this degree of lying the general practitioner finds it hard at times to keep patience, and yet he gains nothing by cursing it. His policy is to accept it and to learn to spot it when he meets with it just as readily as he detects a simple lesion. The obvious cheat, the man who comes to try a fall between experience and deceit, will be easily unmasked. He bears his blushing traces too fairly to deceive any but the tyro. But the man who cannot speak the truth is not so readily recognised, except when he lies painfully and with a stammer—and few patients slur their vowels when they hide their past by discussing their present. The danger is always that we may be tempted to treat such as deliberate perverters, forgetting that there is a psychological basis for their behaviour, and that the lie of the truthfully incapable is a pathological lesion.

We are becoming more tolerant of psychological lesions, and the general practitioners should be in the van in educating the public to bear with them. In his pioneering propaganda he will be able to work with a more honest heart and more determined spirit if he recollects that he, too, is daily bearing in patience the many trials entailed by the untruthfulness of patients.

